# Supplementary material for: Listening forward: approaching marine biodiversity assessments using acoustic methods
Source: R Soc Open Sci. 2020 Aug 26;7(8):201287. doi: 10.1098/rsos.201287 (PMC7481698; doi:10.1098/rsos.201287)
Supplement: List of Terms of Reference [file rsos201287supp1.doc]

**IQOE Working Group on Acoustic Measurement of Biodiversity on Coral Reefs**

### Background

Assessing biodiversity within particular habitats is one of the most difficult challenges faced by biologists. There is a particular need at this time to assess biodiversity, as it is one of the main parameters likely to be affected by the habitat degradation that we are currently experiencing in marine environments around the world.

Coral reefs provide many ecosystem services of value to humankind. Many other invertebrates, fishes, and marine mammals live in close association with the corals, resulting in extremely high productivity and biodiversity. Coral reefs are often referred to as ‘the Tropical Rainforests of the Oceans’. These various organisms form complex food webs, and contribute to human nutrition through their high production of fish and edible invertebrates. However, many coral reefs are currently in a state of decline.

Coral reefs are often subjected to overexploitation by fishermen, increased sedimentation and nutrient overloading, and exposure to high levels of noise from boats and other sources. Natural forces which can cause damage to coral reefs include violent storms, flooding, high and low temperature extremes, El Niño events, and outbreaks of predators and epizootics. Coral reefs are found in shallow tropical waters, along the shores of islands and continents. The reef substrate is mainly composed of calcium carbonate from living and often long-dead corals. Coral reefs are currently threatened by climate change and by other forces. When the water is too warm, UV levels too high, and reefs are exposed to other stressors, corals expel the algae living in their tissues, causing the corals to turn completely white, “coral bleaching”. Nearly all the world’s major coral reef regions (Caribbean/western Atlantic, eastern Pacific, central and western Pacific, Indian Ocean, Arabian Gulf, Red Sea) have experienced some degree of coral bleaching during recent years, with dramatic effects upon biodiversity levels.

Long-term monitoring of coral reefs is required to allow scientists, conservationists, and environmental managers to understand the main forces acting upon coral reefs and resulting in a loss of biodiversity. Currently, the methods for assessing biodiversity rely on catching animals or examining them through visual surveys, often carried out by divers. Visual observations tend to target a few easily observed species and cannot be performed over long time periods or continuously. They seldom take place under dark conditions at night, when many of the organisms living on the reefs are most active. Reef surveys are also expensive and logistically difficult to perform. The results of such surveys may be biased due to the effect of human presence on marine organisms. New methods for assessing biodiversity are needed which are passive, do not disrupt the environment, can be carried out continuously by day and by night, and can cover large areas. Passive acoustic monitoring (PAM) offers a new and flexible way of carrying out such surveys without actually affecting the environment adversely.

Many marine animals—including mammals, fishes and invertebrates—produce sounds as part of their natural behavior. Their calls, or vocalizations, are often species-specific and can be used to confirm the presence of particular animals at a location. Moreover, the calls of such animals are often related to particular activities, including reproductive behavior, and can indicate the status and condition of particular populations. Monitoring the sounds made by marine species, and the soundscapes generated by marine communities, provides a powerful method for assessing biodiversity without having to capture or photograph the organisms. Passive acoustic monitoring of particular habitats, and the development and application of sophisticated acoustical metrics, provides a way of analyzing soundscape structure and relating it to local biodiversity, and in essence a remote sensing method.

Establishment of an IQOE Working Group, to bring together acousticians and ecologists, would take us some way towards developing appropriate methods for monitoring biodiversity in an efficient and non-intrusive way, covering large areas and capable of being applied over long time scales. Such methods would enable us to investigate the effects of human activities upon particular habitats and to monitor the effects of climate and other changes upon biological communities.

Passive acoustic monitoring methods could be applied to a wide range of habitats around the globe. However, in the first instance it would be especially appropriate to apply them to coral reefs; which are amongst the most charismatic and ecologically important habitats found around the world. Yet, it is not currently known how acoustic or soundscape measurements can be applied to adequately assess biodiversity and biodiversity changes. This working group would be aimed at supporting the application of passive acoustic measurements to quantify biota on coral reefs, including how these measurements can provide information about biodiversity and community structure.

### Terms of Reference

The objectives of such a Working Group might be to develop work programmes aimed at the following actions:

- Develop standardized passive acoustic recording protocols for identifying, quantifying, detecting and cataloguing the sounds made by marine organisms and determining the best approaches to provide a coral reef library of sounds.
- Further develop passive acoustics as an ecological survey technique, capable of comparing the acoustic characteristics of different adjacent habitats. This could include initial questions like “what are the relevant spatial and temporal time scales to make such assessments?”
- Develop a suitable analytical technique to identify and quantify vocalizations from marine animals, and the contexts in which they are produced.
- Evaluate and develop analytical techniques that may be used to track and monitor acoustical and community biodiversity over time, techniques that may be able to monitor and inform changes to reefs under stress.
- Determine how changes in the acoustic environment over space and time, especially as a result of human-made noise/sound, affects animals (distribution patterns, migratory behavior and acoustic communication) associated with coral reefs and adjacent habitats their distribution patterns, migratory behavior and acoustic communication.
- Investigate effective sound metrics to characterize the coral reef biodiversity.
- Understanding of the interrelationship among acoustic parameters with respect to the environmental parameters such as temperature, flow, wind data etc.
- Develop pilot projects to apply acoustic techniques to examine the main forces acting upon coral reefs and resulting in changes in biodiversity.
- Generate and leverage support for coral reef soundscape measurements, accurately promoting the utility (and limitations) to managers and other users.
- Help coordinate and stimulate national efforts on the bioacoustics of coral reefs.

The work of the group will be reported in the peer-reviewed literature, at scientific meetings related to bioacoustics and coral reef studies, and through publicly accessible forums.

### 
